# Cell type specificity of female lung cancer associated with sulfur dioxide from air pollutants in Taiwan: An ecological study

**DOI:** 10.1186/1471-2458-12-4

**Published:** 2012-01-04

**Authors:** Ching-Yu Tseng, Yi-Chia Huang, Shih-Yung Su, Jing-Yang Huang, Cheng-Hsiu Lai, Chia-Chi Lung, Chien-Chang Ho, Yung-Po Liaw

**Affiliations:** 1 Doctoral Program in Physical Education, Taipei Physical Education College, Taipei City 11153, Taiwan, R.O.C; 2 Department of Physical Education, Fu Jen Catholic University, New Taipei City 24205, Taiwan, R.O.C; 3 School of Nutrition, Chung Shan Medical University, Taichung City 40201, Taiwan, R.O.C; 4 Department of Public Health and Institute of Public Health, Chung Shan Medical University, Chien-Kuo N. Road, Taichung City 40201, Taiwan, R.O.C; 5 Department and Graduate Institute of Physical Education and Health, Taipei Physical Education College, Taipei City 11153, Taiwan, R.O.C; 6 Department of Family and Community Medicine, Chung Shan Medical University Hospital, Taichung City 40201, Taiwan, R.O.C

**Keywords:** Lung cancer, Squamous cell carcinoma, Adenocarcinoma, Sulfur dioxide, Age-standardized incidence rate

## Abstract

**Background:**

Many studies have examined the association between air pollutants (including sulfur dioxide [SO_2_], carbon monoxide [CO], nitrogen dioxide [NO_2_], nitric oxide [NO], ozone [O_3_], and particulate matter < 10 μm [PM_10_]) and lung cancer. However, data from previous studies on pathological cell types were limited, especially for SO_2 _exposure. We aimed to explore the association between SO_2 _exposure from outdoor air pollutants and female lung cancer incidence by cell type specificity.

**Methods:**

We conducted an ecological study and calculated annual average concentration of 6 air pollutants (SO_2_, CO, NO_2_, NO, O_3_, and PM_10_) using data from Taiwan Environmental Protection Administration air quality monitoring stations. The Poisson regression models were used to evaluate the association between SO_2 _and age-standardized incidence rate of female lung cancer by two major pathological types (adenocarcinoma [AC] and squamous cell carcinoma [SCC]). In order to understand whether there is a dose-response relationship between SO_2 _and two major pathological types, we analyzed 4 levels of exposure based on quartiles of concentration of SO_2_.

**Results:**

The Poisson regression results showed that with the first quartile of SO_2 _concentration as the baseline, the relative risks for AC/SCC type cancer among females were 1.20 (95% confidence interval [CI], 1.04-1.37)/1.39 (95% CI, 0.96-2.01) for the second, 1.22 (95% CI, 1.04-1.43)/1.58 (95% CI, 1.06-2.37) for the third, and 1.27 (95% CI, 1.06-1.52)/1.80 (95% CI, 1.15-2.84) for the fourth quartile of SO_2 _concentration. The tests for trend were statistically significant for both AC and SCC at *P = *0.0272 and 0.0145, respectively.

**Conclusion:**

The current study suggests that SO_2 _exposure as an air pollutant may increase female lung cancer incidence and the associations with female lung cancer is much stronger for SCC than for AC. The findings of this study warrant further investigation on the role of SO_2 _in the etiology of SCC.

## Background

Many studies have examined the association between air pollutants (including sulfur dioxide [SO_2_], carbon monoxide [CO], nitrogen dioxide [NO_2_], nitric oxide [NO], ozone [O_3_], and particulate matter < 10 μm [PM_10_]) and lung cancer [1-5]. However, data from previous studies on pathological cell types were limited. The smoking rate (about 3-4%) in women remained low and stable over the past 30 years [6], but the incidence of female lung cancer was high in Taiwan. It seems that risk factors other than smoking may play a more important role in the pathogenesis of female lung cancer. Our previous results showed that the air pollutants CO [7] and NO [8] had a positive significant association with incidence of lung adenocarcinoma (AC) in Taiwan.

SO_2 _is a common chemical exposure in the pulp and paper product industries, and the concentration often exceeded 2 ppm [9]. SO_2 _is also a major air pollutant and is suspected of increasing the mortality of respiratory diseases in the general population [10-13], and may act as a promoter or a co-carcinogen [14]. In a study of Bond et al. [15], a significant dose-response association was observed between lung cancer mortality and SO_2 _exposure in chemical workers who were exposed to SO_2_. The American Cancer Society Study [16] and the Adventist Health Study [17,18] suggested a strong association between SO_2 _exposure as an air pollutant and increased lung cancer mortality. Higher SO_2 _exposure has been shown to increase lung cancer mortality for both sexes in Abbey's study [17], and an epidemiological study by Lee and colleagues [19] have also been observed. The significant results between SO_2 _exposure and lung cancer mortality was also observed in Asian populations [5,20].

Most of studies have been done to study the association between lung cancer mortality and SO_2 _exposure, with few studies focusing on the association between lung cancer incidence and SO_2 _exposure. Ohyama et al. [21] reported an increased lung cancer incidence in rats exposed to SO_2_. In Beeson's cohort study [18], the relationship between long-term concentrations of ambient air pollutants and risk of incident lung cancer in nonsmoking California adults was evaluated. Results showed lung cancer incidence was positively associated with interquartile range increases for SO_2 _(relative risk [RR] = 1.21; 95% confidence interval [CI], 1.36-3.37) in women. A population-based case-control study was conducted in Stockholm, the result showed a slight association between SO_2 _and male lung cancer incidence [3]. One cohort study also showed non-significant relationship between lung cancer incidence and SO_2 _in Norwegian men [22]. In Vineis's study [23], a nested case-control study was performed in Europe, showing that a non-significant elevated risk was associated with 10 μg/m^3 ^increases of SO_2 _exposure. A recent Dutch cohort study [24] also showed that there was no significant association between SO_2 _and lung cancer incidence. It seems that the association between SO_2 _exposure and lung cancer is still highly controversial. In this study, we focus on the association between SO_2 _and female lung cancer. We conducted an ecological study to explore the association between SO_2 _exposure of air pollutants and female lung cancer incidence by cell type specificity, which might provide new insights for future studies.

## Methods

The air pollution data available for this study were openly obtained from 60 air quality monitoring stations in different municipalities, established by Taiwan Environmental Protection Administration (TEPA) in July, 1993. The 60 monitoring stations were fully automated and provided daily readings of concentrations of air pollutants from 1994 to 1998, including of SO_2_, CO, NO_2_, NO, O_3_, and PM_10_. We calculated annual average of 6 air pollutants (SO_2_, CO, NO_2_, NO, O_3_, and PM_10_) by the TEPA's air quality monitoring stations in these analyses.

The lung cancer incidence data, restricted to females aged older than 30 years in 60 municipalities, were obtained from National Cancer Registration Program (NCRP) operated by the Taiwanese government and collected for the cohort from January 1, 2001 to December 31, 2005. We excluded patients younger than age 30 because the characteristics of early-onset lung cancer are thought to be different from later-onset lung cancer. Smoking is the major confounding factor of lung cancer and there is stable and low smoking rate for female in Taiwan, and so male patients were excluded to minimize the confounding by smoking in this ecological study. The computerized database of the NCRP released by the Department of Health (DOH) of Taiwan for public research purposes and contained the information on date of birth, gender, date of diagnosis, township of residence, and clinical and pathological diagnoses of each patient. All researchers who wish to use the NCRP and its database are required to sign a written agreement declaring that they do not intend to attempt to obtain information that could potentially violate the privacy of patients. This study was evaluated and approved by the DOH for analysis (Application and Agreement Number: 0980300864). Both clinical and pathological diagnoses were coded using the ninth revision of the International Classification of Diseases for Oncology.

To evaluate the possible cell type specificity of the carcinogenic effect of SO_2 _from air pollutants on lungs, two major pathological types (AC and squamous cell carcinoma [SCC]) in Taiwan were considered. Age-standardized incidence rates (ASR) of AC/SCC type cancer were calculated for females. In order to understand whether there is a dose-response relationship between SO_2 _and AC/SCC type cancer, we analyzed 4 levels of exposure based on quartile of concentrations of SO_2_. We used Poisson regression models to evaluate the association between female lung cancer by AC/SCC type cancer and SO_2_. RR and 95% CI from the analyses were adjusted for age and the other air pollutants (including CO, NO_2_, NO, O_3_, and PM_10_). In all analyses, values of *P *< 0.05 were considered statistically significant.

## Results

Table 1 shows that incident cases and age-standard incidence rates of female total lung cancer, AC, and SCC based on the quartiles of concentration of each pollutant. There were 4733 cases of total lung cancer, 2975 cases of AC, and 456 cases of SCC for females were registered in Taiwan from 2001 to 2005. Higher SO_2 _concentrations were associated with higher ASR for SCC.

**Table 1:** Distribution of female total lung cancer, AC and SCC from 2001 to 2005 by quartiles of air pollutants between 1994 and 1998^a^

Variables	All cases^b^		AC		SCC	
	**No. of cases**	**ASR**	**No. of cases**	**ASR**	**No. of cases**	**ASR**

SO_2 _concentration (ppb)						

≤ 3.74	854	37.35	513	22.98	70	2.93

3.74 - 5.52	1209	40.20	785	26.25	114	3.71

5.52 - 7.75	1280	39.96	834	26.09	127	3.99

> 7.75	1390	41.12	843	25.23	145	4.37

CO concentration (ppm)						

≤ 0.55	574	38.35	336	22.84	64	4.29

0.55 - 0.72	736	36.33	466	23.29	65	3.36

0.72 - 0.88	1338	39.81	825	24.84	132	4.12

> 0.88	2085	41.85	1348	27.31	195	3.72

NO_2 _concentration (ppb)						

≤ 17.09	619	38.02	364	22.70	62	3.90

17.09 - 22.18	900	38.92	554	24.47	91	3.85

22.18 - 27.63	1176	39.24	732	22.46	124	4.19

> 27.63	2038	41.21	1325	27.08	179	3.60

NO concentration (ppb)						

≤ 5.29	726	38.43	430	23.53	74	3.96

5.29 - 8.18	727	37.99	433	22.93	81	4.30

8.18 - 12.37	1199	38.15	765	24.51	121	3.82

> 12.37	2081	42.27	1347	27.65	180	3.66

O_3 _concentration (ppb)						

≤ 19.18	1932	43.08	1252	28.39	174	3.85

19.18 - 22.01	1208	37.13	761	23.45	123	3.87

22.01 - 25.33	822	37.93	502	23.31	81	3.74

> 25.33	771	39.83	460	24.54	78	3.96

PM_10 _concentration (μg/m^3^)						

≤ 50.04	1302	39.28	814	25.06	109	3.22

50.04 - 61.23	1362	40.83	884	26.67	126	3.72

61.23 - 76.30	1168	42.88	737	27.35	119	4.42

> 76.30	901	36.23	540	22.09	102	4.16

^a ^AC, adenocarcinoma; ASR, age-standardized incidence rate; CO, carbon monoxide; NO, nitric oxide; NO_2_, nitrogen dioxide; O_3_, ozone; PM_10_, particulate matter < 10 μm; SCC, squamous cell carcinoma; SO_2_, sulfur dioxide.

^b ^All cases included AC, SCC, large cell carcinoma, and small cell carcinoma.

Table 2 shows the results of the Poisson regression analyses, which the dependent variables were the ASR of female total lung cancer, AC, and SCC, and SO_2_, CO, NO_2_, NO, O_3_, and PM_10 _were independent variables. Using SO_2 _concentration ≤ 3.74 ppb as the baseline, the RR for lung cancer among females at 3.74 < SO_2 _≤ 5.52 ppb was 1.09 times (95% CI, 0.97-1.22) higher than the baseline level, 1.10 times (95% CI, 0.97-1.24) at 5.52 < SO_2 _≤ 7.75 level, and 1.30 times (95% CI, 1.13-1.50) when SO_2 _> 7.75 ppb. When we further analyzed whether higher SO_2 _concentrations indeed correspond to higher risk of lung cancer, there was a significant dose-response relationship between SO_2 _concentrations and the risk of lung cancer (*P *= 0.0003).

**Table 2:** Poisson regression analyses for female total lung cancer, AC and SCC from 2001 to 2005 based on quartiles of air pollutants between 1994 and 1998^a^

Variables	All cases^b^	AC	SCC
	**RR^† ^(95% CI)**	**RR^† ^(95% CI)**	**RR^† ^(95% CI)**

SO_2 _concentration (ppb)			

≤ 3.74	1.00	1.00	1.00

3.74-5.52	1.09 (0.97-1.22)	1.20 (1.04-1.37)	1.39 (0.96-2.01)

5.52-7.75	1.10 (0.97-1.24)	1.22 (1.04-1.43)	1.58 (1.06-2.37)

> 7.75	1.30 (1.13-1.50)	1.27 (1.06-1.52)	1.80 (1.15-2.84)

Test for trend	*P *= 0.0003*	*P *= 0.0272*	*P *= 0.0145*

CO concentration (ppm)			

≤ 0.55	1.00	1.00	1.00

0.55-0.72	0.97 (0.84-1.12)	0.99 (0.83-1.19)	0.82 (0.53-1.27)

0.72-0.88	1.14 (0.98-1.32)	1.09 (0.90-1.31)	1.07 (0.69-1.67)

> 0.88	1.20 (0.99-1.45)	1.18 (0.93-1.50)	0.99 (0.56-1.75)

Test for trend	*P *= 0.0055*	*P *= 0.0759	*P *= 0.5833

NO_2 _concentration (ppb)			

≤ 17.09	1.00	1.00	1.00

17.09-22.18	0.93 (0.82-1.05)	1.06 (0.90-1.25)	0.95 (0.64-1.41)

22.18-27.63	0.87 (0.75-1.01)	1.08 (0.89-1.30)	1.08 (0.69-1.70)

> 27.63	0.80 (0.66-1.08)	1.15 (0.89-1.47)	1.04 (0.57-1.91)

Test for trend	*P *= 0.0304*	*P *= 0.3175	*P *= 0.7259

NO concentration (ppb)			

≤ 5.29	1.00	1.00	1.00

5.29-8.18	1.11 (0.97-1.26)	1.05 (0.89-1.24)	1.05 (0.71-1.57)

8.18-12.37	1.02 (0.88-1.17)	1.13 (0.94-1.36)	1.04 (0.67-1.62)

> 12.37	1.27 (1.05-1.53)	1.31 (1.03-1.66)	1.12 (0.63-1.99)

Test for trend	*P *= 0.0615	*P *= 0.0174*	*P *= 0.7464

O_3 _concentration (ppb)			

≤ 19.18	1.00	1.00	1.00

19.18-22.01	0.82 (0.75-0.91)	0.84 (0.75-0.95)	0.79 (0.59-1.06)

22.01-25.33	0.86 (0.77-0.97)	0.85 (0.73-0.98)	0.82 (0.57-1.16)

> 25.33	0.89 (0.78-1.02)	0.88 (0.74-1.04)	0.75 (0.50-1.14)

Test for trend	*P *= 0.1169	*P *= 0.1188	*P *= 0.2049

PM_10 _concentration (μg/m^3^)			

≤ 50.04	1.00	1.00	1.00

50.04-61.23	1.08 (0.98-1.19)	1.03 (0.92-1.15)	1.12 (0.82-1.52)

61.23-76.30	1.17 (1.06-1.30)	1.07 (0.95-1.20)	1.34 (0.98-1.83)

> 76.30	0.99 (0.88-1.12)	0.85 (0.73-1.01)	1.22 (0.84-1.78)

Test for trend	*P *= 0.3893	*P *= 0.1700	*P *= 0.1413

^a ^AC, adenocarcinoma; CI, confidence interval; CO, carbon monoxide; NO, nitric oxide; NO_2_, nitrogen dioxide; O_3_, ozone; PM_10_, particulate matter < 10 μm; RR, relative risk; SCC, squamous cell carcinoma; SO_2_, sulfur dioxide.

^b^All cases included AC, SCC, large cell carcinoma, and small cell carcinoma.

^†^Adjusted for age and other air pollutants. *P < 0.05.

We further stratified the lung cancer incidence data based on two major cell type specificity to investigate the association between SO_2 _from air pollutants and the risks of AC and SCC among females. With increasing SO_2 _concentrations, the RR of AC went up 1.20, 1.22, and 1.27 times of the baseline level, and that of SCC went up 1.39, 1.58, and 1.80 times of the baseline level (Table 2). The dose-response relationships of RR for AC and SCC existed for AC (*P *= 0.0272) and SCC (*P *= 0.0145). The other five air pollutants (CO, NO_2_, NO, O_3_, and PM_10_) showed significant RR only at level NO > 12.37 ppb (RR, 1.31; 95% CI, 1.03-1.66) for AC, and the five air pollutants are all non-significant for SCC.

## Discussion

Our results showed that two (SO_2_, NO) of six air pollutants (SO_2_, CO, O_3_, NO, NO_2_, and PM_10_) are significantly associated with female lung incidence for AC, but only SO_2 _among six air pollutants was significantly associated and achieved a dose-response relationship with female lung incidence for SCC. This is consistent with our previous paper which showed that the air pollutants NO [8] are positive significant association with incidence of AC in Taiwan. To the best of our knowledge, this ecological study was the first to focus on the relationship between cell type specificity of female lung cancer and SO_2 _from outdoor air pollution by using the cancer register data from 2001 to 2005 in Taiwan. The interesting and new finding is that the association of female lung cancer is much stronger for SCC than for AC in this study although the SCC only comprising 9.63% of female lung cancer while AC comprises 62.86% of female lung cancer in Taiwan.

In contrast with our results, Nyberg et al. [3] and Nafstad et al. [22] indicated non-significant associations between SO_2 _exposure and male lung incidence. Additionally, both Vineis's [23] and Beelen's study [24] also showed non-significant association between lung cancer incidence and SO_2 _exposure even though gender was adjusted. The discrepancy between our results and previous studies [3,22-24] might be because the cell type specificity of lung cancer was not considered in the previous studies. In addition, gender might directly interact with SO_2 _exposure and lung cancer incidence rather than just play a role as a confounder. In agreement with Beeson's cohort study [19], there was a significant association between SO_2 _exposure and lung cancer incidence. Moreover, we further observed the association between SO_2 _exposure and SCC was stronger than the association between SO_2 _exposure and AC.

The association between exposure to the SO_2 _and the generation of human lung AC and SCC can be used for assessment of effect using "dose-response relationship" and "biological plausibility" criteria. For "dose-response relationship" criteria, with increasing SO_2 _concentrations, the RR of AC went up 1.20, 1.22, and 1.27 times of the baseline level, and that of SCC went up 1.39, 1.58, and 1.80 times of the baseline level. The dose-response relationships of RR for AC and SCC were shown for AC (*P *= 0.0272) and SCC (*P *= 0.0145) in this study. For "biological plausibility" criteria, bronchial epithelial cells are the progenitor cells for bronchogenic lung cancers [25], and often exposed to airborne environmental pollutants such as SO_2_. SO_2 _is readily absorbed through the respiratory tract (99%) and subsequently dissociates to form its derivatives (bisulfate and sulfite in neutral fluid) [26]. The data in Qin's study [27] supported the hypothesis that SO_2 _derivatives could cause the inactivation of tumor suppressor genes and SO_2 _derivatives may play a role in the pathogenesis of SO_2 _associated lung cancer. SO_2 _in air pollution may play an important role in the reaction of epidermal growth factor receptor (EGFR). High dose of SO_2 _would increase the EGFR expression in human bronchial epithelial cells [28]. EGFR mutation target the peripheral airway and give rise to lung AC [29,30]. Therefore, high SO_2 _exposure in the environment might potentially increase AC incidence for lung cancer, and significantly test for trend (*P *= 0.0272) has been observed at Table 2 in the present study. Environmental SO_2 _may also increase the activity of tumor suppressor p53 (TP53) gene in human bronchial epithelial cells [27]. TP53 has been shown to be related to the risk of SCC [31-33]. Although we did not tend to discuss TP53 in this study, this may explain why high SO_2 _exposure increased the risk of SCC and significantly test for trend (*P *= 0.0145) at Table 2 in the present study.

It is difficult to study the association between urban air pollution and lung cancer in prospective studies. A major challenge is the assessment of individual long term air pollution exposure in prospective cohort studies. Therefore, long term air pollution exposure on an aggregated (non-individual) level has been assessed to study the association between air pollution and lung cancer in most of studies [2,16,34-38].

However, three important issues in our ecological study should be mentioned. First, smoking is well-known potential confounders when lung cancer is studied. Unfortunately, there was a lack of information on smoking in the present study. Though it has been shown that smoking was unlikely correlated with air pollution levels [39], the likelihood of confounding varies from place to place. We analyzed the distribution of air pollutants in the 60 municipalities between 1994-1998 and found that geographical variations in SO_2 _(mean, 6.33 ppb; coefficient of variation [CV] %, 64.42) and NO concentrations (mean, 9.54 ppb; CV%, 65.43) were higher than the other air pollutants (data not shown). We also showed the correlations between the various pollutants and found the correlation coefficient is 0.46 (data not shown). This implied SO_2 _could be interpreted as an indicator of industrial air pollution (rather than air pollution like NO from traffic); and there may well be some relationship also between smoking habit and residence (or not) in a more industrialized municipality. To minimize the residual confounding of smoking, we only study female lung cancer incidence since women had a lower and stable smoking rate (2.9-5.3% in 1972-2008) when compared to male smoking rate in Taiwan [6]. The lower female smoking rates may result in the smaller difference of lung cancer incidence between municipalities based on the floor effect. Therefore, the lower female smoking rates might not be the major contributor for female lung cancer in the present study, there might be another contributor like SO_2 _other than smoking to cause female lung cancer in Taiwan.

Second, the role of environmental tobacco smoke (ETS) playing in the causation of lung cancer has been evaluated since 1981 [40]. Despite many epidemiological studies showing that a significantly increased nonsmoking female lung cancer risk is associated with ETS exposure [41-43], most of these studies have been conducted in Western countries (i.e., the United Status and Europe). An the Asia-Pacific Chinese region, a meta-analysis combining six studies in nonsmoking Chinese women showed no excess risk related to ETS, with an overall OR of 0.91 (95% CI = 0.75-1.10) [44]. This was reinforced in two case-control studies in Hong Kong [45] and Taiwan [46]. These two studies reported that exposure to ETS was not a significant risk factor for lung cancer among nonsmoking females. However, another study conducted in Southern Taiwan reported positive association between ETS and risk of female lung cancer [47]. The inconsistent results could partially be explained by the cell type specificity of lung cancer. Some studies suggested that passive smoking was a risk factor for lung cancer, particularly of AC in Hong Kong Chinese women who never smoked [48]. Another study in Russia found that the association between exposure to ETS of the spouse and risk of lung cancer in non-smoking women was non-significant for both SCC and AC [41]. The lung cancer study in China for ever exposed to ETS from spouse was also non-significant [49]. A prospective study in Japanese non-smoking women found that passive smoking was a risk factor for lung cancer, especially for AC among Japanese women [43]. Although ETS may well be a risk factor for lung cancer, there is currently no strong or clear evidence indicating ETS is a risk factor for lung SCC in non-smoking women in Asia. We, therefore, assume that ETS is unlikely to have been an important confounder in the present study and not controlling for ETS is unlikely to have had an important effect on our results.

**Figure 1:**
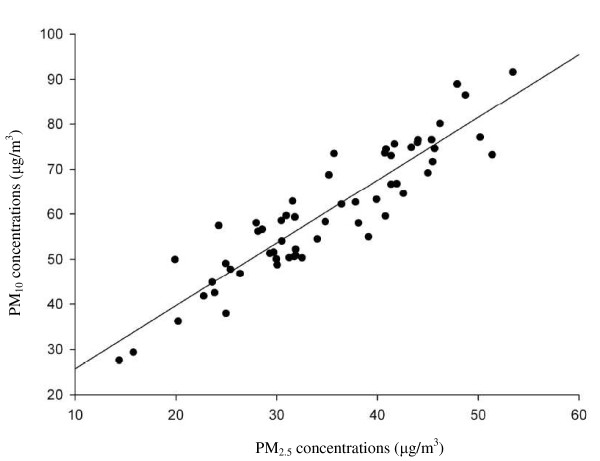
**Correlation analyses between PM_10 _and PM_2.5 _from 60 township monitoring stations in 2006**.

Third, Pope et al. [35] found an 8% increases in lung cancer mortality for each 10 μg/m^3 ^increase in particulate matter < 2.5 μm (PM_2.5_) concentrations in the American Cancer Society Cancer Prevention Study II. An extend analysis of the Harvard Six Cities Study also found a positive association between PM_2.5 _and lung cancer mortality [50]. It is worth noting that these two cohort studies [35,50] only used annual average PM_2.5 _concentrations as the air pollution exposure index, not simultaneously considered the effects of other air pollutants (i.e., SO_2_, NO, NO_2_, O_3_, and PM_10_) on the observed association; and the measure of effect was mortality rather than incidence. However, the air pollution monitoring stations of Taiwan could only provide the data on PM_10 _rather than PM_2.5 _during the study period (1994-1998) since the TEPA did not measure PM_2.5 _concentrations until 2006. Although we could not discuss the association between PM_2.5 _and female lung cancer incidence in 1994-1998, there was a strong positive correlation (*r *= 0.91, *P *< 0.0001) between PM_10 _and PM_2.5 _among the 60 monitoring stations from 2006 data in Taiwan (Figure 1). This implies the similar geographic variation for both PM_10 _and PM_2.5 _across these monitoring stations in 2006. The air pollution levels during the study period (1994-1998) could be a reasonable indicator of historical trend over the past 20 to 30 years [7]. We, therefore, assumed that there would be a similar SO_2 _effect on female lung cancer incidence after adjusting either PM_2.5 _or PM_10_. We incorporated PM_10 _into the Poisson regression model for analyses, and found no association between PM_10 _and two main cell type specificity of female lung cancer incidence. In addition to PM_2.5_, we noticed that the sulfur oxide-related pollution was also associated with lung cancer in the study of Pope et al. [35]. Previous studies showed the association of lung cancer mortality was a non-significant for PM_2.5 _but significant for SO_2 _[51,52]. Although the positive trend was observed between PM_2.5 _concentrations and lung cancer incidence in an ecologic study [53], the low R^2 ^(< 0.10) implied that other etiologic agents rather than PM_2.5 _might affect lung cancer incidence. Therefore, it could not be ruled out that SO_2 _is a potential risk factor for lung cancer even though there might be an association between lung cancer incidence and PM_2.5_.

## Conclusions

The current study suggests that SO_2 _exposure as an air pollutant may increase female lung cancer incidence and the association of female lung cancer is much stronger for SCC than for AC. The findings of this study warrant further investigation on the role of SO_2 _in the etiology of SCC.

## Abbreviations

AC: Adenocarcinoma; ASR: Age-standardized incidence rates; CI: Confidence interval; CO: Carbon monoxide; CV: coefficient of variation; DOH: Department of health; ETS: Environmental tobacco smoke; NCRP: National Cancer Registration Program; NO: Nitric oxide; NO_2_: Nitrogen dioxidel; O_3_: Ozone; PM_2.5_: Particulate matter < 2.5 μm; PM_10_: Particulate matter < 10 μm; RR: Relative risk; SCC: Squamous cell carcinoma; SO_2_: Sulfur dioxide; TEPA: Taiwan Environmental Protection Administration.

## Competing interests

The authors declare that they have no competing interests.

## Authors' contributions

LYP participated in the design and conducted the study, interpreted the results and helped to draft and edit the manuscript. TCY and HCC participated in the design and conducting the study and also contributed to the writing of the revised manuscript. HYC participated in the design of the study and helped to revise the manuscript. SSY and HJY participated in the data analysis. LCH helped in conducting the study. LCC helped to revise the manuscript. All authors read and approved the final manuscript.

## Pre-publication history

The pre-publication history for this paper can be accessed here:

http://www.biomedcentral.com/1471-2458/12/4/prepub
